# Cholera Infantum: Its Cause and Treatment

**Published:** 1898-09

**Authors:** 


### CHOLERA INFANTUM: ITS CAUSE AND TREATMENT.

Doan, in *Pediatrics* of August 15, 1898, says that for the most part the diseases of the first two years of life are the acute infectious ones, and that cholera infantum is an acute milk infection. The perfectly normal man is proof against germs. He may swallow with impunity the cholera germ, the typhoid bacillus, or any other germ, so long as the alimentary canal is intact. The gastric juice of a healthy stomach is ordinarily able to destroy all germs that enter therein, while the bile acts as a safeguard for the intestines. So long as these protective agencies are in active operation, the body retains its ability to resist the attacks of the microbes. A normal infant is thus protected by its gastric juice from the ravages of germ life. It is true that the gastric secretion is scant, and its germicidal power is weak, but Nature does not intend for the infant to crowd the stomach or to fill it with food swarming with bacteria. No factor enters so largely into the causation of disease in infants as does excessive and improper feeding. It is evident from the severity of cholera infantum, the great wasting of the child, and its suddenness and fatality, that it is due to some dread poison or gastro-intestinal irritant. The bowel is found to swarm with bacteria, all of which are busy liberating their ptomaines, varying only in their intensity.

Acute or chronic intestinal indigestion may be given as an etiological factor in cholera infantum, but the author thinks that these troubles are as independent as tuberculosis and la grippe. It is a poison either introduced with food or liberated by chemical reaction within the alimentary canal. Factors favoring such are heat of summer, bottle-feeding, infectious milk, uncleanliness of bottle and nurses, and excessive and improper feeding. To recognize the trouble as an acute poisoning instead of a case of catarrhal inflammation will reduce the dreadful rate of mortality. To give calomel and then regulate the bowels was on the right plan—that is, to help Nature free the alimentary canal of the poison—but it is too slow. In fact, all cathartics are too slow, allowing too much time for the poison to be absorbed. We must act upon the idea that the toxic material exerts its influence by causing great depression of the heart and the system generally by acting on the nerve centers and by paralyzing the vasomotor nerves of the intestines. Taking this view we have the following indications to meet: (1) Assist Nature in emptying the stomach and bowels; (2) neutralize or overcome the effect of the poison on the heart and nervous system; (3) reduce the temperature. To do the first we must thoroughly irrigate the bowels and wash out the stomach with sterilized water. For the second indication nothing equals morphine and atropine in minute doses For the third indication, the injection of normal salt solution

The frequent injection into the buttock in varying amounts has proven to be very useful. To reduce the fever phenacetine has been given, as well as the other coal-tar products, with good results. The diet for several days should be limited, of a bland and easily digested food.—*Medical Age.*

